# Can collaborative orientations strengthen or weaken effectiveness of improvisers’ emergency response to an emergency incident? A conditional process model

**DOI:** 10.3389/fpsyg.2025.1547414

**Published:** 2025-04-28

**Authors:** Tu JunMei, Xu Zhengquan

**Affiliations:** ^1^College of Xu Hai, China University of Mining and Technology, Xuzhou, China; ^2^School of Management, China University of Mining and Technology, Xuzhou, China

**Keywords:** cognitive appraisals, emergency response, improvisational ability, behavioral orientations, crisis setting

## Abstract

Due to insufficient information on the on-site emergency incident, respondents face a huge challenge to react effectively. This may suggest that the individual response to emergency tasks is likely to differ from that in daily work contexts. This study aims to explore the conditional process of how individuals’ improvisational cognitive appraisals (IICAs) in psychology affect their improvisational performance in an emergency setting and its consequences. Using the data garnered from the coal mine accident rescue teams we find that the level of IICAs positively impacts the individual’s improvisational ability to effectively respond to the emergency incident and the improvisational performance of temporary emergency teams as a whole. Further, an individual’s higher level of proclivity to seek cooperation with others in an emergency situation weakened the relationship between the individual’s improvisational cognitive appraisals and the individual’s improvisational performance team. Our work sheds light on how the improvisational performance of temporary emergency teams in a crisis setting is shaped, explaining why those who perform well in their day-to-day work often have difficulties in achieving the same good performance in a sudden crisis setting.

## 1 Introduction

Improvisation is a critical capability for individuals being in an emerging crisis situation (Kim, 2021; Mannucci et al., 2021; Weick et al., 1999). Individuals in such situation need to go beyond just following procedures and executing strategic plans (Miner et al., 2001), otherwise they will face exacerbation of the crisis, because individuals need to respond quickly to ongoing crisis events and the situation so that the crisis can be prevented and controlled (Rudolph et al., 2009). Individual’s improvisation is a spontaneous process, in which planning and execution happen simultaneously (Crossan, 1998; Crossan and Sorrenti, 2003), which can make the difference between death and survival, both metaphorically (Brown and Eisenhardt, 1997) and literally (Bechky and Okhuysen, 2011; Weick, 1993, 1996). Research documented that the performance of temporary organizations to respond to rapidly changing crises mainly depend on their employees’ ability to improvise (Leberecht, 2016; Patriotta and Gruber, 2015). Although an extensive and rapidly growing body of existing literature has investigated the critical roles that individual’s improvisation functions in promoting effectiveness of the emergency and fast-response team (Livne-Tarandach and Jazaieri, 2021), yet little is known about the mechanism of how IICAs affecting their improvisational ability and performance of temporary emergency teams (Cutuli, 2014; Jacques et al., 2024). Some of the scholars have explored factors influencing the relations between an individual’s improvisational ability and cognitive appraisals (Bechky and Okhuysen, 2011; Patriotta and Gruber, 2015; Vera and Crossan, 2005), and find that individuals who can fast cognitively respond to the emerging crisis show high cognitive appraisals and improvisational ability (Faraj and Xiao, 2006). To date, it is still difficult to use experimental means to explore how their improvisational ability to cope with crisis develops and corroborate whether the successful improvisational behavior performed in the previous emergency situation can repeat in a different emergency scene (Rudolph et al., 2009). Further research reveals that individuals show obvious differences in the formation of the improvisational ability (Rudolph, 2003). In addition to formal training, Cognitive appraisal theory suggests that individuals also can learn improvisation skills through practice (Hardy and Maguire, 2020; Karunakaran, 2022; Myers, 2022). Since the process of developing individuals’ improvisational ability is very complex, scholars call for further studies of how individuals develop their improvisational ability, and what factors affect this process (Sutton et al., 2021).

Based on the above arguments, we develop a conditional process model to explain how individuals’ improvisational ability can influence the improvisational performance of temporary emergency teams that they affiliate with. This model shows the relations between antecedents and effects of individuals’ improvisation in crisis setting, as well as the conditions on which those relations rely. Further, drawing on the concept of cognition in psychology (Jacques et al., 2024), we propose a new concept of the improvisational cognition, the concept suggests that individual with cognitive ability and skills know how to deal with crisis and take effective action. Therefore, we argue that individual’s higher level of improvisational ability in an emergency situation largely depends on the level of their improvisational cognitive appraisals.

Data were collected from series of interviews with the members of coal mines rescue teams (CMRT), we know that members of CMRT are not always regular. When a new accident happens, the leaders and members of the CMRT may change accordingly. Therefore, the CMRT shows obvious characteristics of temporary organizations. During the rescue operations, the tasks performed by each member are changing, and the collaboration between them is also not constant over time. When the accident happens, the CMRT needs to be formed in a very short time in order to launch rescue operations immediately; thereby the CMRT will consist of incumbent members, as well as some new comers. This means that it will be very difficult for such team to develop cooperative ability in a short period of time. In addition, during the rescue operations, the CMRT may encounter some unexpected novel risks that they unknown, so members of the CMRT need to make adaption over time in such emergency situation. Thus, when the team’s collaborative ability is difficult to form in a short period of time, the individual members’ improvisational ability will become particularly important. Therefore, CMRT is an ideal context for our data collection.

Our research shows that the development of mine accident rescue teams’ performance in emergency setting is a conditional process. First, when an emergency incident happens, rescue members must first develop the ability to identify the existence of risks in an extremely short time, which is a prerequisite for the development of an individual’s improvisational skills. Further research shows that mine rescue teams’ performance in emergency settings relies on individuals’ improvisational skills. If rescue team members lack improvisational ability, it will be much more difficult for them to deal with the ongoing risks and the emergency environment in which the crisis is embedded, implying that emergency rescue team cannot achieve higher performance. In addition, our research also shows that due to the uncertainty and danger of emergency crises, the actions taken by rescue team members in emergency setting are difficult to supervise, and their responsibilities in such environment is difficult to clarify and quantify too. This may lead them to shirk responsibility for the purpose of self-protection when performing urgent tasks that need cooperation. If this is the case, they may leave tasks with greater risks to their coworker, while taking on low-risk or no-risk ones as much as possible. Therefore, in an emergency setting, the effect of IICAs on their improvisational ability and the performance of temporary emergency teams that the individuals situate in is largely conditioned by level of their proclivity to seek cooperation with others.

## 2 Theory and hypotheses

### 2.1 Cognitive appraisals and its impact on individual’s improvisational skills

Cognition in the psychology refers to the process by which knowledge and understanding is developed in the mind of individuals. Literature shows that the level of individuals’ cognitive appraisals has an impact on their activities (Bell, 2012). Vergne and Depeyre (2016) found that the effectiveness of individual’s response to the emergency crisis largely depends on their knowledge of the crisis (Vergne and Depeyre, 2016). Chandler et al. (2020) demonstrates that the low level of individuals’ cognitive appraisals can make them unaware of the omen of pending disasters in the emergency setting. Thus, when the disaster happens, individuals with low level cognitive appraisal are unable to respond effectively, or may react in a wrong way, and cannot capture the best chance to cope with the ongoing crisis (Chandler et al., 2020), which may lead to escalation of the crisis. Therefore, individuals’ improvisational cognitive appraisal ability is of key importance to their effective response to the unfolding disaster in an emergency crisis setting.

Based on information processing theory, cognitive is the ability of the human brain processing information (Burke et al., 2006; Sackett, 1998). Further study shows that the higher cognitive ability, the greater the ability of individuals to analyze and deal with problems in the emergency setting (Lebel and Patil, 2018). According to the theory of the expectancy confirmation biases, individuals often dismissed external information that is not in favor of their own ideas (Lord et al., 1979; Staw, 1981). Therefore, the individuals’ gathered information in an emergency situation largely relies on their cognitive bias (Wry and York, 2017), they may pay little attention to unimportant or irrelevant information according to their own cognitive bias (Hambrick and Mason, 1984; Richard et al., 2021). Then they will dedicate sufficient time and effort to analyze and process such information to support their decision in the emergency setting (England, 1967; Jost et al., 2003; Wry and York, 2017).

According to Jost et al. (2018) and Sternberg (1985), individual’s ability depends on his cognitive level (Jost et al., 2018; Sternberg, 1985). When outcomes of individuals improvisational actions meet or even exceed their expectations in an emerging crisis situation means that their improvisational ability is high. The same logic holds that the improvement of individuals’ cognitive appraisal will promote their improvisational ability and the subsequent performance. Gutwin and Greenberg (2004) shows that learning is a key way to improve cognitive ability (Gutwin and Greenberg, 2004). The level of individuals’ cognition is the first thing that actors can change in an emergency crisis environment. Thus, we can posit that individuals’ improvisational ability in a crisis setting improve with the increase of the cognitive level. Further research shows that cognitive ability determines whether actors can clearly see the root cause of the crisis and quickly find a solution to resolve it. From the perspective of cognitive logic, one’s cognitive appraisal directly determines his improvisational ability in a crisis setting, which in turn affects the overall emergency performance of the temporary emergency teams. Additionally, the theory of collective behavior (Granovetter, 1978) suggest that the overall emergency performance of the temporary emergency teams as a whole is the compounding of individual emergency response capabilities. Therefore, we can assume:

Hypothesis 1 (H1): The level of an individual’s cognitive appraisals will have a direct positive impact on his improvisational ability in an emergency crisis setting.

Hypothesis 2 (H2): There is a positive correlation between an individual’s cognitive appraisals and the overall emergency performance of the temporary emergency teams as a whole.

### 2.2 Improvisational ability and its impacts

Mintzberg (1973) introduced the concept of improvisation into management research domain to explain how some strategies emerge adaptively as a response to ongoing environmental changes (Mintzberg, 1973; Mintzberg and Waters, 1985). The effective strategy should keep up with the change in the environment (Mannucci et al., 2021). Later, Weick (1993), 642) offered the formal definition of improvisation. Under such definition, when investigating the deaths of 13 firefighters in the infamous Mann Gulch fire disaster, he note that the three survivors, relying on a “burst of improvisation,” were able to escape the fire as a result of their ability to think on their feet and act quickly (Mannucci et al., 2021; Weick, 1993). To our knowledge, fire scene does not repeat, firefighters may encounter some novel emergency crisis setting and issues in enacting fire rescue tasks every time. If this is the case, the firefighters’ improvisation will be very important for them to effectively respond to the ongoing fire disaster. This landmark work by Karl Weick inspired further research on how improvisation works in context characterized by a lack of predictability due to frequent surprises and time pressure (Mannucci et al., 2021). In this research, we refer to such a context as the “emergency crisis setting” and argue that emergency crisis context is lack of predictability. Findings from these settings show that improvisations directed toward solving emergency issues and creating novel outcomes are not mutually exclusive; on the contrary, they can coexist (Mannucci et al., 2021).

Further, from the notion of improvisation, the relationship between an individual’s improvisational ability and the emergency performance of the temporary emergency teams also can be discerned (Lehrer, 2012). When the execution and crafting of the plan almost concurrently occurs, then we can say that activity of planning and execution is improvisational. Improvisation is a spontaneous response to unplanned events that helps individuals to solve problems or find new ways in response to unknown events (Barrett, 1998; Kamoche and Cunha, 2001; Moorman and Miner, 1998a, 1998b; Vera and Crossan, 2004; Vera and Crossan, 2005; Weick, 1993). We use this concept to examine the reactive, spontaneous action taken by individuals in an emergency setting. Research shows that in an emergency setting, performance of a temporary organization depends on the degree to which improvisational planning matches improvisational execution.

Scholars agree on some of the core elements of the definition of improvisation, but they do not agree on how to improve individual ability to improvise. Individual’s ability to improvise may take many years to develop (Lehrer, 2012). The mechanism of training individuals’ improvisational skills is very complex (Darr et al., 1995; Gino et al., 2010). If we want to improve the improvisational performance of temporary rescue teams, we need to understand the mechanism of the development of individuals’ improvisational ability, then we can know how to use it to achieve the goal in the emergency settings. Therefore, some scholars advise that if temporary organizations want to improve their improvisational emergency performance, they first need to improve their members’ improvisational abilities (Eisenberg, 1990; Olson, 1989). Accordingly, I predict,

Hypothesis 3 (H3): There is a positive relationship between individuals’ improvisational ability and the improvisational performance of temporary rescue teams in which they situate.

#### 2.2.1 Mediating role of individuals’ improvisational ability

The mechanism of the development process of individuals’ improvisational ability affecting the improvisational performance of temporary rescue teams in which these individuals situate is very complex (Eisenhardt and Tabrizi, 1995). Individuals’ cognitive appraisal needs to be transformed into their improvisational ability in order to have a positive impact on the improvisational performance of temporary rescue teams (Vera and Crossan, 2005). Based on the above discussion, we know that changes of the level of individuals’ cognitive appraisals will have a direct impact on individuals’ improvisational ability and the improvisational performance of temporary rescue teams in the emergency settings. We can infer that the individuals’ improvisational ability in crisis setting mediates the effect of their cognitive appraisal on the improvisational performance of temporary rescue teams as a whole. Therefore, we may posit that the impact of the level of IICAs on the performance of temporary rescue teams may be partially mediated through their improvisational ability. Therefore, we may posit:

Hypothesis 4: Individuals’ improvisational ability partially mediates the impact of the level of individuals’ improvisational cognitive appraisal on the performance of temporary rescue teams.

### 2.3 The moderating effect created by the collaborative orientation

Ozcan and Gurses (2018) shows that interaction between members of a group exhibits some degree of proclivity or bias (Ozcan and Gurses, 2018), which can be divided into two categories, namely cooperative orientation and competitive orientation, respectively. Individuals’ orientation can help improve their job performance. Consistent with this logic, individuals can either choose to cooperate with or compete with others to improve their own improvisational performance when they work in the same emergency crisis setting (Cronin and Weingart, 2007; De Dreu and Boles, 1998; Weingart et al., 2007). Further, resources invested in enacting urgent tasks are scarce in an emergency crisis setting, which makes individuals who engage in urgent tasks have to compete for these scarce resources. The competition occurring among emergency respondents can lead to negative consequences (Nalick et al., 2020).

Although research on improvisation assumes that collaboration among team members is largely inherent in the execution of emergency tasks (Barrett, 1998; Peplowski, 1998), yet this is not always the case. When emergency event occurs, the team leaders required to building efficient emergency rescue team before the rescue operation starts. The leaders of the team usually motivate everyone to think and act in the same way, when they are situating in the crisis setting (Bezrukova et al., 2009; Kane, 2010; Vestal and Danneels, 2022). Before the start of the rescue operation, the purpose of kick-off meeting is to motivate the newly established rescue team members to pursue a common goal so as to avoid problems provoked by a small portion of members who want to stand out by displaying greater virtuosity that may undermine the team’s performance (Mannucci et al., 2021). With the evolution of the emergency rescue operation, some members’ actions may deviate from the team’s goal, which will result in the failure of team coordination and cooperation. However, forming new effective cooperation among team members and developing effective sense making that facilitates the exploitation of the connections between members need to consume attention and time in the emergency environment (Vera et al., 2014).

Therefore, if the individual members of the temporary rescue team focus their attention on seeking cooperation with other peers in an emergency crisis setting, their attention invested in foster ability to improvise will be at risk of being crowded out. For individuals immersed in emergency situations, their attention is a scarce resource, and which has a major impact on their emergency decision making (Clough and Piezunka, 2020; Joseph and Ocasio, 2012; Ocasio, 1997; Piezunka and Dahlander, 2015; Sullivan, 2010). Therefore, the more they invest such resources in seeking cooperation with others, the less attention he can devote to developing ability to improvise, as will potentially hinder the formation of their improvisational ability. Further research shows that adverse effect of individuals’ negative evaluation of collaboration within social structures of the temporary rescue team on the performance of temporary rescue teams as a whole does not hold constant. When the magnitude of individuals’ collaborative orientation changes, the effect of the level of IICAs on their improvisational ability and the performance of temporary rescue teams may change accordingly. Thus, we can offer the following assumptions:

Hypothesis 5 (H5): Individuals’ collaborative orientation negatively moderate the impact of IICAs on their improvisational ability. When individual’s unwillingness to collaborate increase, the impact will be weakened.

Hypothesis 6 (H6): Individuals’ collaborative orientation negatively moderate the impact of IICAs on the performance of temporary rescue teams. When individual’s unwillingness to collaborate increase, the impact will be weakened.

**Figure 1:**
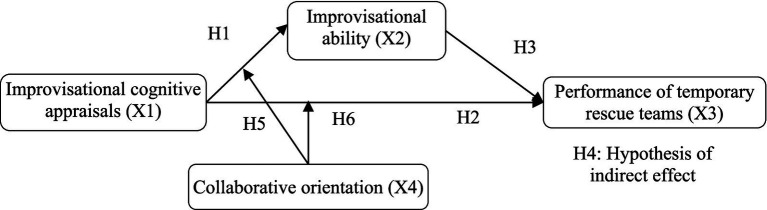
Theoretical model.

According to these theoretical assumptions, we build the following hypothetical relationship network in Figure 1.

## 3 Research methodology

### 3.1 Data and sample

We choose the CMRT as our research context. In China, CMRT need to deal with various emergency occurring in coal mining operation in their daily work. Although each coal mining firm has its own accident rescue team, the members of the team are not always fixed. Perhaps in each execution of coal mining accidents’ rescue tasks, new members may join the team and incumbent members may depart, this will make the CMRT show salient characteristics of a temporary organization. Moreover, a large number of coal mine accident rescue cases show that even if emergency plans for accidents are well prepared, they may not be fully implemented in emergency setting. Because during an accident rescue operation, new crises and emergency tasks will frequently occur, so it is impossible for these teams to fast-draft emergency plans for coping with these new crises and emergency tasks. Thus, members need to improvise when performing emergency tasks, and complete implementation of the prescribed emergency plan may imply catastrophic consequences. In this research, we choose the employees of the coal mine accident rescue teams as research sample and use self-reported questionnaires to measure the constructs.

#### 3.1.1 Samples and data

The data were obtained through interviews and questionnaires with rescues team members. The survey was carried out in period of two months form June 1, 2022 to July 31, 2022. A total of 339 rescue team members from 25 coal mining enterprises in 3 major coal-producing provinces were interviewed. The questionnaires were designed with short and simple sentences to ensure that respondents could complete the questionnaires within 15 min. 294 questionnaires were returned, of which 11 is not completed, and be removed. Finally, we got 283 qualified questionnaires. The qualified response rate of the questionnaires was 83.5%. The demographic characteristics of those respondents included age (mean = 36.430; sd = 8.230), gender, level of education and magnitude of crisis experience accumulation (“A” denotes no “crisis experience,” B means “little crisis experience accumulation,” “C” indicates “rich crisis experience accumulation,” see Table 1).

**Table 1:** The demographic characteristics of the respondents (*N* = 283).

Controls	Term	Frequency	Percentage
Sex	Male	207	57.7%
	Female	152	42.3%
Age	≤20	22	6.1%
	21 ~ 30	278	77.4%
	31 ~ 40	34	9.5%
	≥41	25	7.0%
Education level	Else	14	3.9%
	High school	222	61.8%
	College and Undergraduate	106	29.5%
	postgraduate	17	4.7%
Crisis experience	A	91	25.3%
	B	67	18.7%
	C	201	56.0%

### 3.2 Measures

#### 3.2.1 Level of IICAs (X1)

In line with Prem et al. (2017), we develop a 4-item scale to measure the individuals’ cognitive appraisal level in the emergency crisis setting. These four-point Likert scale (ranked as 1 = strongly disagree to 5 = strongly agree) is: “I can figure out the cause of an emergency crisis within a very short time “; “I can figure out what action to take in response to the current crisis within a very short time “; “I can create an emergency plan to deal with the crisis within a very short time “, “I can predict the changes of the ongoing crisis, so I can adoptively change the emergency plan to effectively respond to the ongoing crisis.” The Cronbach’s *α* for this scale was 0.707.

#### 3.2.2 Individuals’ improvisational ability (X2)

In line with Vera and Crossan (2005), we developed a 5-point Likert scale to measure Individuals’ improvisational ability in emergency crisis setting. The 5-point scale (ranking as 1 = completely disagree to 5 = completely agree) contains four items, namely “I can respond quickly when I face with emergency incidents,” “I can create emergency plans and execute them almost simultaneously when I am assigned emergency tasks,” “I can effectively react in right way when the emergency incident unfolds,” “I believe that I hold very good improvisational adaptability.” The Cronbach’s *α* for this scale was 0.808.

#### 3.2.3 The performance of temporary rescue teams (X3)

We measured performance of temporary rescue teams by assessing the effectiveness of their execution of urgent tasks in the emergency crisis setting. Following Baer et al. (2021) and Banin et al. (2016), we used a five-item scale to measure the performance of temporary rescue teams (X3-5): “We believe that our temporary rescue teams can best perform ever emergency task,” “Now, our temporary rescue teams are very good at performing emergency tasks,” “Overall, I deem that our temporary rescue teams are excellent at emergency collaboration and coordination,” “After each rescue operation, in most cases, we may be praised by our upper superiors.” “After each rescue operation, we are rarely criticized by our upper superiors.” The Cronbach’s α for this scale was 0.794.

#### 3.2.4 Individuals’ collaborative orientation (X4)

According to Mannucci et al. (2021), Individuals’ collaborative orientation will influence their willingness to seek collaboration and help from other peers. In emergency crisis settings, due to the scarce, limited resources, individuals’ who hold competitive orientations may be reluctant to provide help and collaboration to the one who needs them. In line with Wang and Zatzick (2019), we develop a three-item scale to measure the level of individuals’ collaborative orientation in emergency crisis settings. For ease of interpretation, what we measure is the level of an individual’s unwillingness to collaborate (X4) in the crisis setting. Each of the 5-point (1 = completely disagree to 5 = completely agree) scale items are: “When I am doing the urgent task, at the same time, a new unexpected urgent task emerges, I am reluctant to consult other team members on how effectively responding to the new urgent task, because I deem that doing so will slow down my response speed.” “When I am doing the urgent task, but a new unexpected urgent task emerges, I generally do not ask other team members for help.” “When I am doing the urgent task, but a new unexpected urgent task emerges, it is generally impossible for me to get substantial help from other members, so when other members seek help from me, I do not provide substantial help either.” The Cronbach’s α for this scale was 0.830.

### 3.3 Control variables

Factors that affect the human’s improvisational ability in emergency crisis setting are various and complex. Prior evidence has shown that individual’s ability to improvise is linked to gender (Seedat et al., 2009), age (Kessler et al., 2007), education (Fryers et al., 2003), and experience accumulating via executing urgent tasks (Stansfeld et al., 2011) influence human’s improvisational ability in emergency crisis setting. Thus, we controlled for emergency respondents’ age, gender, education level, and magnitude of experience accumulating via executing urgent tasks.

Gender was coded as 1 for women and 0 for men. Education was coded as: “1” for else, “2” for a high school, “3” for four-year university degree, and “4” for master degree (Kim and Kim, 2020). The age of the respondents was coded as:1 if age ≤ 20, 2 for age “21 ~ 30,” 3 for age “31 ~ 40,” and 4 for age “≥41.” We control for magnitude of experience accumulating via executing urgent tasks, we code “A” for no crisis experience, “B” for little crisis experience accumulation, and “C” for rich crisis experience accumulation.

## 4 Results and analyses

### 4.1 Reliability and validity of measurement

Reliability and validity are used to evaluate the consistency and accuracy of the measurement, respectively. We evaluated the factor structure of the measures through a confirmatory factor analysis (CFA) of the four latent variables in the hypothesized model (Alinejad and Anvari, 2019; Campbell and Fiske, 1959; Jackson, 1977). Fornell and Larcker (1981) proposed that the average extracted variation (AVE) is used to estimate convergent and discriminant validity (Fornell and Larcker, 1981). We used a multilevel confirmatory factor analysis (MCFA) to evaluate the factor structure of the latent variables in our model by using Mplus8.3 (Schilpzand et al., 2018).

As shown in Table 2, the standardized loading in the measurement model were high and load on their respective factors. Interval of the factor loading ranges between 0.561 to 0.883. The hypothesized four-factor model, in which individual scale items loaded on separate first-order latent factors, displayed good fit ($\chi^{2}(98)$= 138.185, *p* = 0.0047; RMSEA = 0.038; SRMR = 0.036; CFI = 0.978) (Hu and Bentler, 1999). The baseline model, in which all the standardized loading load on one of the four factors, provided bad fit for the data ($\chi^{2}(120)$=1939.024, *p* = 0.0000). We further performed CFA to examine the hypothesized model with 3-factor and 2-factor, and the results show that the 4-factor model has the best fit. Results suggested that the four-factor model fitted the data better than all others.

**Table 2:** Results of the confirmatory factor analysis.

Latent variable	Items	Standardized Loadings	SE	*P*-value	C.R.	AVE
X1	x11	0.654	0.040	0.000	0.7114	0.4821
	x12	0.561	0.044	0.000		
	x13	0.603	0.044	0.000		
	x14	0.650	0.039	0.000		
X2	x21	0.779	0.029	0.000	0.811	0.5185
	x22	0.658	0.038	0.000		
	x23	0.707	0.034	0.000		
	x24	0.731	0.032	0.000		
X3	x31	0.782	0.029	0.000	0.8015	0.5497
	x32	0.569	0.045	0.000		
	x33	0.662	0.038	0.000		
	x34	0.616	0.041	0.000		
	x35	0.704	0.035	0.000		
X4	x41	0.772	0.033	0.000	0.8349	0.6294
	x42	0.716	0.036	0.000		
	x43	0.883	0.028	0.000		

Table 2 shows that the convergent validity (C.R.) of the five latent variable is between 0.7114 and 0.8349, and the value of AVE is between 0.4821 and 0.6294. Only the AVE of the first factor is slightly lower than 0.5. Therefore, in the 4-factor model, scales of all 4 unobserved variables have acceptable convergent validity. The higher reliability of the scale and the higher validity of the conceptual model ensure that the following hypothesis tests can be based on reliable data.

Table 3 provides the descriptive statistics including the means, standard deviations, correlations of the variables, and reliability estimates.

**Table 3:** Descriptive statistics, correlations, and reliabilities.

Variables	Mean	SD	X1	X2	X3	X4	Age	Sex	Edu	Experience
X1	3.660	1.089	(0.707)							
X2	3.726	1.147	0.668**	(0.808)						
X3	4.126	1.000	0.690**	0.715**	(0.794)					
X4	1.689	0.861	0.307**	0.359**	0.247**	(0.830)				
age	36.430	8.230	0.027	0.001	0.03	0.139				
sex	0.950	0.224	−0.099	−0.06	−0.116	−0.104	−0.014			
edu	1.840	0.735	−0.024	0.002	0.049	0.006	−0.015	0.055		
region	2.090	0.878	−0.085	−0.022	−0.075	0.016	0.078	−0.011	−0.01	

*p < 0.05, **p < 0.01 and Cronbach’s αs in parentheses with the diagonal.

### 4.2 Test for direct effect

Table 4 shows that, using 5,000 bootstrap samples, a 95% confidence interval of the correlation coefficient between IICAs (X1) and individuals’ improvisational ability (X2) ([0.9561 to 1.4022]) is positive and significant (B = 1.1792, *p* = 0.0000), does not include zero. Thus, the proposed Hypothesis 1 is supported. The results indicates that, in emergency settings, the higher level of IICAs (X1), the higher individuals’ improvisational ability (X2) will be.

**Table 4:** Path coefficients of the conceptual model.

	Consequent							
	X2				X3			
Antecedent		Coeff	SE	p		Coeff	SE	p
X1	$\alpha_{1}$	1.1792	0.1133	0.0000	$c_{1}^{\prime }$	0.4830	0.1102	0.0000
X2					$c_{2}^{\prime }$	0.4147	0.0495	0.0000
Constant	i_M1_	−0.9903	0.4667	0.0347	i_M2_	0.9030	0.4694	0.0554
	$R_{x2}^{2}$=0.4875				$R_{x3}^{2}$= 0.5478			
	$F_{x2}(4,278)$= 66.0975, *p* < 0.05				$F_{x3}(8,274)$= 41.4932, *p* < 0.05			

$\alpha_{1}$ denotes the effect of X1 to X2; $\;c_{1}^{\prime }$ denotes the effect of X_1_ on X3; $c_{2}^{\prime }$ denotes the effect of X2 on X3.

Table 4 shows that individuals’ improvisational ability (X2) also has a direct positive effect on performance of temporary rescue teams (X3) (B = 0.4147, *p* = 0.0000). The confidence interval [0.3172, 0.5121] is positive and significant, does not include zero, suggests that the higher individuals’ improvisational ability (X2) is, the higher performance that temporary rescue teams (X3) shows, thus support Hypothesis 3. Further, Table 4 shows that IICAs (X1) also have a direct impact on performance of temporary rescue teams (X3) (B = 0.4830, *p* = 0.0000). The confidence interval [0.2661,0.7000] is positive and significant, does not include zero. Thus, Hypothesis 2 is also supported.

### 4.3 Test for conditional indirect effect (X1 → X2 → X3)

Table 5 provides the results of the moderation mediation hypotheses (H5 and H6) by using the PROCESS macro for SPSS (Hayes, 2017).

**Table 5:** Conditional indirect effects of X1 on X (X1 → X2 → X3).

X4	Effect	BootSE	BootLLCI	BootULCI
1.0000	0.3469	0.0511	0.2534	0.4534
1.6890	0.2491	0.0392	0.1721	0.3245
2.5501	0.1268	0.0566	0.0011	0.2217

**Figure 2:**
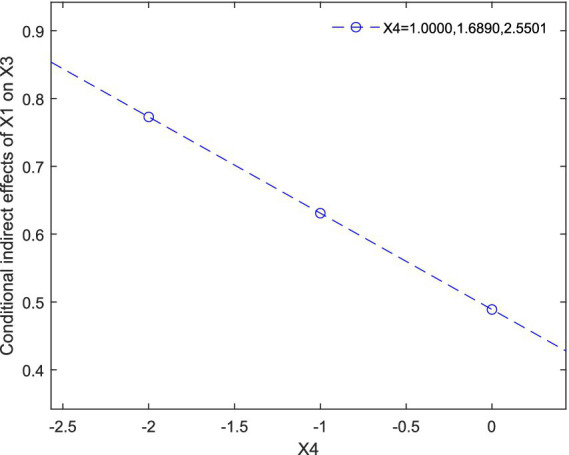
Conditional indirect effect of X1 on X3.

In Figure 2 we assigned values to X4 as 1.0000, 1.6890, 2.5501, respectively, to test the conditional indirect effect of X1 on X3.

The Table 5 shows that, using 5,000 bootstrap samples, when X5 is assigned values of 1.0000, 1.6890, 2.5501, respectively, all 95% bootstrap confidence intervals (Boot LLCI, Boot ULCI) do not straddle 0 and include negative values, which suggests that the conditional indirect effect of IICAs (X1) on the performance of temporary rescue teams (X3) is positive and significant. Further, the results also indicates that as the value of X4 increases, the conditional indirect effect gradually decreases. Such effect can be explained by the reinforcement sensitivity theory (DeCelles et al., 2020; Gray, 1976; Grey and McNaughton, 1982; McNaughton and Gray, 2000). Thus, the Hypothesis 4 is supported. Figure 2 graphically shows that this conditional indirect effect is decreasing with increases of individuals’ collaborative orientation.

### 4.4 Test for moderating effect

Table 6 shows the results to test hypotheses about conditional indirect effects after controlling for emergency respondents’ age, gender, education level, and magnitude of experience accumulating via executing urgent tasks. The standardized regression coefficient for “X1 × X4 “(B = -0.3425) is negative and significant (*p* = 0.0000), a 95% confidence interval ([0.4808 to −0.2042]) does not include zero. Therefore, we can conclude that the effect of IICAs (X1) on the individuals’ improvisational ability (X2) is negatively moderated by individual’s unwillingness to collaborate (X4). Thus, Hypothesis 5 was supported (see Table 7).

**Table 6:** Results from a regression analysis examining the effect of X1 on X2 moderated by X4.

Model	Coeff	se	t	p	LLCI	ULCI
Constant	−0.9903	0.4667	−2.1220	0.0347	−1.9090	−0.0716
X1	1.1792	0.1133	10.4068	0.0000	0.9561	1.4022
X4	1.5161	0.3000	5.0535	0.0000	0.9255	2.1067
Int_1	−0.3425	0.0703	−4.8748	0.0000	−0.4808	−0.2042
Region	0.0296	0.0564	0.5243	0.6005	−0.0814	0.1405
R^2^ = 0.4875, F(4, 278) = 66.0975, *p* = 0.000 Int_1: X1 × X4						

**Table 7:** Results from a regression analysis examining the effect of X1 onX3 moderated by X4.

Model	Coeff	se	t	*p*	LLCI	ULCI
Constant	0.9030	0.4694	1.9238	0.0554	−0.0211	1.8270
X1	0.4830	0.1102	4.3828	0.0000	0.2661	0.7000
X2	0.4147	0.0495	8.3736	0.0000	0.3172	0.5121
X4	0.5376	0.2588	2.0772	0.0387	0.0281	1.0471
Int_1	−0.1375	0.0604	−2.2753	0.0237	−0.2564	−0.0185
Age	0.0036	0.0050	0.7238	0.4698	−0.0062	0.0135
Sex	−0.2910	0.1828	−1.5916	0.1126	−0.6509	0.0689
Edu	0.0770	0.0554	1.3901	0.1656	−0.0320	0.1861
Region	−0.0498	0.0467	−1.0671	0.2869	−0.1417	0.0421
R^2^ = 0.5478, *F* (8, 274) = 41.4932, *p* = 0.000 Int_1: X1 × X4						

**Figure 3:**
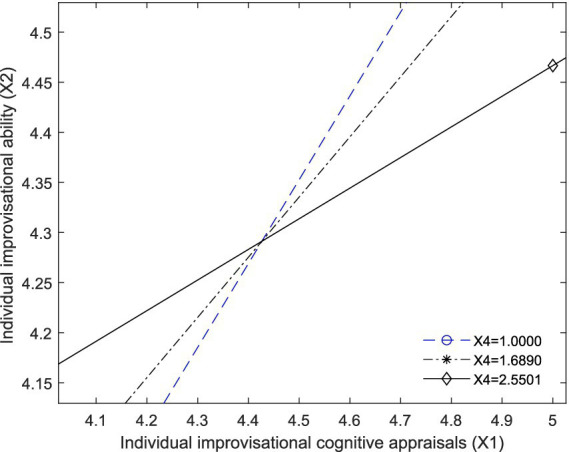
A visual representation of the conditional effects of X1 on X2 among those relatively low (X4 = 1.0000), moderate (X4 = 1.6890), and relatively high (X4 = 2.5501) level of individual’s unwillingness to collaborate (X4).

According to Figure 3, the standardized regression coefficient for “X1 × X4” (B = -0.1375) is negative and significant (*p* = 0.0000), and a 95% confidence interval coefficient ([LLCI, ULCI] = [−0.2564, −0.0185]), does not include zero. These results suggest that individual’s unwillingness to collaborate (X4) negatively moderate the relationship between IICAs (X1) and performance of temporary rescue teams (X3). Thus, the proposed Hypothesis 6 is supported.

**Figure 4:**
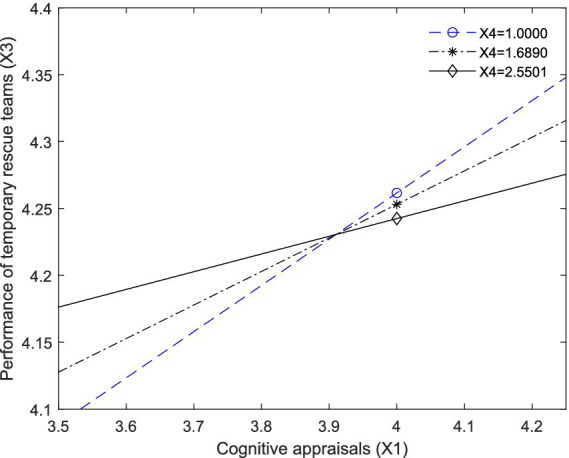
A visual representation of the conditional effects of X1 on X3 among those relatively low (X4 = 1.0000), moderate (X4 = 1.6890), and relatively high (X4 = 2.5501) level of individual’s unwillingness to collaborate (X4).

We also can observe the moderation effect from Figures 3, 4. Figure 3 plots the visualizing effect of X1 on X2 moderated by X4, and Figure 4 plots the visualizing effect of X1 on X3 moderated by X4.

## 5 Discussion

This study aims to better understand the mechanism of IICAs and individuals’ improvisational ability that affecting the performance of temporary rescue teams in emergency crisis settings, so as to help temporary emergency response organizations to improve their improvisational performance. Findings mostly support our proposed hypotheses. Below, we will discuss the theoretical and practical implications of the findings in this study.

### 5.1 Theoretical implications

First, our theoretical model shed light on the different roles played by IICAs and their improvisational ability in influencing performance of temporary rescue teams as a whole. Thus, we expand the literature on improvisation and provides a theoretical framework for understanding how the level of IICAs affects the performance of temporary rescue teams as a whole. To our knowledge, scholars have not drawn a clear line between IICAs and their improvisational ability. In this study, we argue that IICAs (what to do) are different from their improvisational ability (what can do). Individuals who show a very low level of improvisational cognitive appraisals are unlikely to show high ability to improvise in emergency setting.

Second, this research contributes to the literature on the mechanism of how performance of temporary rescue teams evolves. Findings shows that IICAs and their improvisational ability are two critical factors that shape the performance of temporary rescue teams in emergency setting. According to the theory of collective action, in an emergency crisis setting, IICAs and their improvisational ability can combine to affect the performance of temporary rescue teams. The above logic shows that although improvisation of the temporary rescue teams is a sort of spontaneous response, which is determined by IICAs and their ability to improvise.

Third, our findings further shed light on the mechanism by which the effects of IICAs and their improvisational ability on the performance of temporary rescue teams are conditioned. Because the plan-making and plan-implementation occur simultaneously in an emergency crisis setting, this leads to the consequence that the pre-arranged plan is difficult to fully implement. Research reveals that people showing different value orientations may exhibit different levels of collaborative orientation in an emergency crisis setting (Slade Shantz et al., 2020). The coal mine accident rescue operation is actually sort of a collective action, and individual members must interact and communicate frequently while performing emergency tasks. Due to the scarcity of emergency resources, the effects of IICAs and their improvisational ability on the performance of temporary rescue teams are contingent on the degree of collaborative orientation that individuals show in emergency crisis settings.

### 5.2 Practical implications

First, the results of our research have important practical implications for how temporary rescue teams can improve their improvisational ability when facing emergency incidents. In reality, emergency respondents may encounter many so-called surprises for which they cannot find a ready resolution. When this is the case, how to effectively deal with these so-called surprises will be of great significance not only to the emergency respondents themselves but also to the entire temporary rescue teams in which they are situated. Because these unexpected challenges and surprises may not only get emergency respondents into trouble but even endanger their lives. If this happens, other members have to devote extra efforts to rescue the trapped team members, they are unable to continue the execution of emergency tasks at hand, which may adversely affect the entire crisis management process. Therefore, improving individuals’ ability to improvise, they can mitigate crises by deploying their own emergency resources without requiring other members to collaborate or coordinate.

Second, our research has important practical implications for understanding the role of improvisation in emergency crisis settings. The results shows that individuals’ improvisational performance largely depends on their ability on how they improvise in emergency settings. Further, our finding also shows that individuals’ ability to improvise hinges on how they can quickly adopt improvisational cognitive ability. Thus, in the practice, if the individuals can figure out the mechanism of the crisis unfolding, then they will be able to know how to effectively respond to it.

Further, if a temporary organization wants to improve its improvisational performance in an emergency crisis setting, it needs to try to improve the improvisational cognitive appraisals of its individual members. Thus, the findings of this study can help emergency organizations design more feasible training programs for improving the improvisation of their members.

### 5.3 Limitations and future research

First, the data was garnered from coal mine accident rescue teams in China, which will affect the generalization of the results to extend to other emergency crisis settings. Future research needs to explore improvisation in other crisis settings to further confirm the generalization of the findings of this study.

Second, we conclude that cooperation among individual members in emergency crisis settings, can amplify effect of IICAs (X1) and their improvisational ability (X2) on performance of temporary rescue teams (X3). But from the perspective of limited resources, cooperation among individual members may weaken the above effects. This is because, in an emergency crisis setting, although an established emergency plan that has proven effective in the past has helpful in accelerating decision processes, it also has limitations in drafting quick organizational responses. Such discrepancy deserves further research in the future.

Finally, how to measure individuals’ improvisational ability in emergency crisis settings is still an unsolved puzzle. From the cognitive perspective, a person’s cognitive ability to understand and cope with crises is first related to his brain power. However, a person with a lot of brainpower cannot fully release the brainpower to form his cognition in the setting. This means that he does not hold high improvisational ability in emergency crisis settings. Therefore, in emergency crisis settings, individuals’ improvisational ability mainly depends on whether they can make full use of their brain power in a very short time spell. In this study we did not address such issue, future research can focus on it.

## Data Availability

The datasets presented in this study can be found in online repositories. The names of the repository/repositories and accession number(s) can be found in the article/supplementary material.
